# Efficient photocatalytic degradation of organic pollutants over TiO_2_ nanoparticles modified with nitrogen and MoS_2_ under visible light irradiation

**DOI:** 10.1038/s41598-023-35265-7

**Published:** 2023-05-31

**Authors:** Heba M. El Sharkawy, Amira M. Shawky, Rania Elshypany, Hanaa Selim

**Affiliations:** 1 Department of Analysis and Evaluation, Egyptian Petroleum Research Institute, Nasr City, 11727 Cairo Egypt; 2 Sanitary and Environmental Institute (SEI), Housing and Building National Research Center (HBRC), Giza, 1770 Egypt

**Keywords:** Environmental sciences, Chemistry, Nanoscience and technology

## Abstract

Investigate the use of visible light to improve photocatalytic degradation of organic pollutants in wastewater. Nitrogen-doped titania and molybdenum sulfide nanocomposites (NTM NCs) with different weight ratios of MoS_2_ (1, 2, and 3 wt.%) synthesized by a solid state method applied to the photodegradation of methylene blue(MB) under visible light irradiation. The synthesized NTM composites were characterized by SEM, TEM, XRD, FT-IR, UV–Vis, DRS and PL spectroscopy. The results showed enhanced activity of NTM hybrid nanocrystals in oxidizing MB in water under visible light irradiation compared to pure TiO_2_. The photocatalytic performance of NTM samples increased with MoS_2_ content. The results show that the photodegradation efficiency of the TiO_2_ compound improved from 13 to 82% in the presence of N-TiO_2_ and to 99% in the presence of MoS_2_ containing N-TiO_2_, which is 7.61 times higher than that of TiO_2_. Optical characterization results show enhanced nanocomposite absorption in the visible region with long lifetimes between e/h+ at optimal N-TiO_2_/MoS_2_ (NTM_2_) ratio. Reusable experiments indicated that the prepared NTM NCs photocatalysts were stable during MB photodegradation and had practical applications for environmental remediation.

## Introduction

The textile, paper, cosmetics, pharmaceutical, and food industries all make extensive use of colored dyes ^1–3^. Due to definite causes, dye-contaminated water, especially from the textile industry, is challenging to clear. Most of these colored dyes are of synthetic origin and usually consist of aromatic rings in their molecular structure, Inert and non-biodegradable when discharged into waste water without proper treatment^4,5^. Therefore, removing such dyes from polluted water is highly urgent in terms of protecting human health and environmental resources ^6^. Methylene blue (MB), the most commonly used base dye, is believed to have multiple uses in the printing and dyeing industry ^7^. According to a report, the textile industry accounts for about 67% of the dyes market and/or consumption, with 120 cubic meters of industrial wastewater discharged for every ton of fibre produced. Despite the importance of MB in many industries, its presence in the environment and human health can be compromised if not managed effectively. In which MB is carcinogenic and does not degrade due to the characteristic stability of aromatic rings in the molecular structure of MB. Traditional biological, chemical and physical techniques such as adsorption and chemical precipitation are recognized for the treatment of dyeing wastewater. These methods are expensive, form sludge or generate secondary pollutants, such as dye adsorption on activated carbon, where the pollutant only converts from the liquid phase to the solid phase, causing pollution. secondary infection. Accordingly, the decomposition of dyes into non-toxic compounds is essential and recommended ^8–11^. Advanced oxidation processes (AOP) are currently attracting a great deal of attention in the field of water treatment ^12^. To extend the lifetime of photogenerated electron–hole pairs, hybrid photocatalysts composed of semiconductor heterojunctions^14,15^. Semiconductors have been used in AOPs to photocatalytically degrade organic contaminants, especially those with the ability to absorb visible light, due to their band gaps ^13–18^. Among photocatalytic semiconductors, titanium dioxide (TiO_2_) has attracted great interest due to its ability to readily decompose organic pollutants, strong oxidizing ability, low toxicity, chemical stability, low cost, and availability^19,20^. The photocatalytic performance of TiO_2_ is mainly determined by the lifetime of photogenerated electronhole pairs, but the fast recombination rate of electron–hole pairs in TiO_2_ limits its application in photocatalysis^21^.To extend the lifetime of photogenerated electron–hole pairs, hybrid photocatalysts composed of semiconductor hetero-junctions should suppress the fast recombination rate of photogenerated charge carriers ^22,23^. In this regard, many efforts have been made to reduce the band gap and improve its photocatalytic activity ^24,25^. A recognized material to extend the photoresponse range to visible light is to dope TiO_2_ with a non-metallic dopant, nitrogen ^26,27^. The combination of TiO_2_ and nitrogen at different energy levels improves the electron–hole separation efficiency and enhances the efficiency of the photocatalyst reaction. Furthermore, by combining TiO_2_ with other bandgap semiconductors such as MoS_2_, it is possible to create heterogeneous photocatalysts. MoS_2_ is a non-toxic, highly stable, strong oxidizing and relatively inexpensive material. Due to its large surface area, MoS_2_ can act as an excellent catalyst for N-TiO_2_^28,29^. MoS_2_ exhibits a layer-dependent tunable bandwidth, an indirect bandwidth of 1.2 eV, a direct bandwidth of 1.9 eV, and high theoretical catalytic activity ^30,31^. Due to their bandgap, semiconductors have been used in AOPs to photocatalytically degrade organic pollutants, especially those with the ability to absorb visible light.Combining N-TiO_2_ and MoS_2_ at different energy levels improves the efficiency of electron–hole separation and enhances the efficiency of the photocatalytic reaction ^32,33^.The work presented here focuses the visible-light-driven photo degradation of a dye contaminants, specifically methylene blue (MB) dyes, into environmentally friendlyCO_2_ and H_2_O. novel hetero- nanocomposite of N-TiO_2_/MoS_2_ (NTM) as a photocatalyst using a solid-state method with low temperature synthesis, cost efficiency and easy control of reaction kinetics compared other methods. In addition, the physicochemical properties of the obtained samples have been extensively investigated to discover the excellent photocatalytic activity for MB decomposition under visible light radiation compared with pure TiO_2_. The synthesized NTM has proven to be an effective photocatalyst for applications in environmental protection.

## Experimental

### Materials

The chemicals used in this work were: Titanium (IV) isopropoxide (TIPO) [Ti (OCH (CH_3_)_2_)_4_], sodium molybdate (Na_2_MoO_4_), ethylene glycol and thiourea with purity (99.95%) is obtained from petrochemical company, Egypt. NaOH, NH_4_OH, ethanol and nitric acid (HNO_3_) with purity (90–99%) and methylene blue dye were purchased from Merck KGaA (Darmstadt, Germany). All solutions have been prepared using freshly deionized water. And purchased compounds were used as received, without further purification.

### Nanoparticles synthesis

#### Synthesis of TiO_2_ nanoparticles (T)

TiO_2_ NPs were synthesized using sol–gel method ^14^. In a typical synthesis, an appropriate amount of Ti isopropoxide precursor mixed to dis-H_2_O was dissolved in 87.5 ml of ethanol and then stirred for 4 h at room temperature, washed several times with deionized water and ethanol, and then dried in oven at 90 °C over night. Finally, the resulting powder was calcinated at 500 °C in a muffle furnace for 1 h in the air to extract the TiO_2_ NPs.

#### Synthesis of TiO_2_&N nancomposite (NT)

The sol gel method^34^ was used to synthysize N&TiO_2_ (NT) nanocomposite. Firstly, 10 mL TIPO was added to 40 mL ethanol and vigorously stirred at RT for 30 min (solution A). Secondly, (solution B) contains10 mL of ethanol, 10 mL of NH_4_OH solution (28 wt.%), and 2 mL HNO_3_. Then, solution A was added to solution B with solwley addaition under vigorous stirring. The obtained yellow semi-transparent sol was created after 2 h continuous stirring, then aged for 6 h at room temperature in air to form a homogeneous gel, which was dried for 36 h in an electric oven at 80 °C. Finally, the dry gel was milled into powders and calcined at 400 °C for 4 h a furnace set in air with a heating rate of 3 °C min^−1^ to yield NT nanocomposite.

#### Synthesis of MoS_2_ nanoparticle (M)

MoS_2_ nanoparticles (M) were synthesized by a solvothermal reaction^35^. In this process, Na_2_MoO_4_ (3 mmol, 0.726 g) and ethylene glycol (40 mL) were dissolved in 50 mL deionized water, then add thiourea (15 mmol, 1.1418 g). The mixed solution was sonicated for 30 min at RT, transferred to a Teflon lined stainless steel autoclave and kept at 180 °C for 12 h. After cooling to room temperature, the products were separated by centrifugation, washed three times with absolute ethanol and deionized water, and then dried at70 ℃ for 12 h. Finally, the black powder was obtained.

#### Synthesis of N-TiO_2_/MoS_2_ (NTM) nanocomposites

N-doped TiO_2_/MoS_2_ nanocomposites (NTM) were synthesized by a solid state method. The N- TiO_2_/MoS_2_ composite was prepared by using different weight ratios of NT:M (1:1, 1:2, and 1:3 wt%) are labeled as NTM_1_, NTM_2_, and NTM_3_, then milled together, sonicated using a prope sonicator for 15 min, and washed several times then air dried.

### Experimental techniques

The morphology of prepared materials was studied by transmission electron microscope (TEM) model JEM-2100, JEOL, Japan and scanning electron microscope (SEM) (JEOL). The phase of the prepared samples was examined by X-ray diffraction (XRD) using a diffractometer (Panalytical XPERT PRO MPD). CuKα radiation (λ = 1.5418 Å) was used at 40 kV and 40 mA. The functional groups were identified using a Fourier transform infrared (FT-IR) spectrometer model Spectrum One (Perkin Elmer, USA) in the wave number range of 400–4000 cm^−1^. Optical reflectance was recorded using a UV–Vis spectrometer (Perkin Elmer Lambda 1050). The photoluminescence spectra were recorded by a Cary Eclipse fluorescence spectrophotometer.

### Photocatalytic activity study

The photocatalytic degradation activity was investigated using aphotoreactor with 400 W Halogen lamp as the light source. The distance between the halogen lamp and the dye solution is 10 cm. Then, 0.025 g of hetero-photocatalyst was added to 50 mL of 50 ppm MB dye solution and to achieve adsorption–desorption equilibrium, the solution was stirred in the dark for 30 min. The photodegradation reaction was initiated for 150 min, and 5 mL of the suspension was collected a period of 15 min. The obtained suspension was analyzed by UV–vis spectrophotometer at at MB solution maximum absorption wavelength at 664 nm.

## Results and discussion

**Figure 1:**
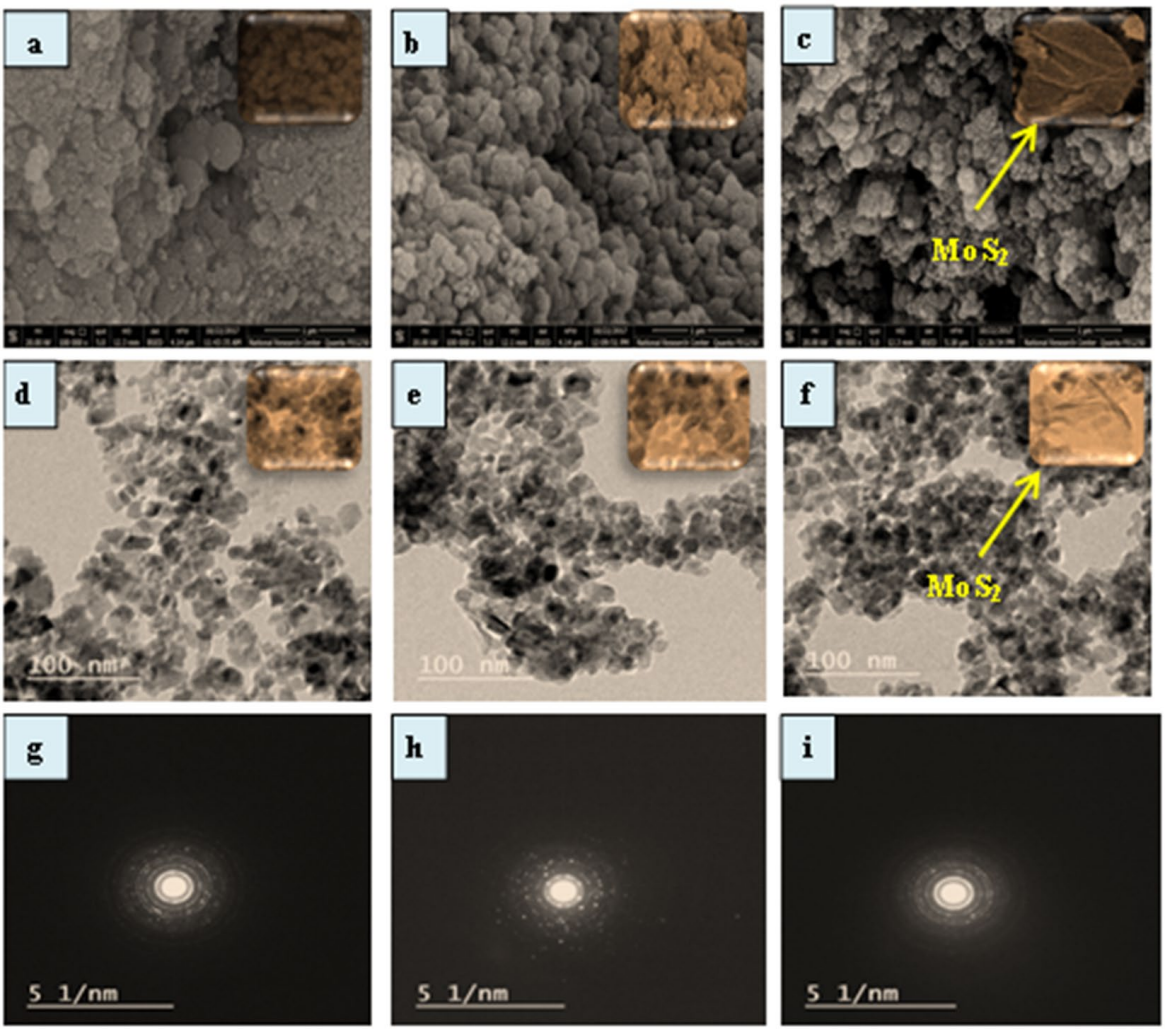
SEM images (**a**–**c**), TEM images (**d**–**f**) of the pure TiO_2_, NT and NTM_2_ and their SAED patterns (**g**–**j**) respectively.

Figure 1 illustrates the SEM images of pure TiO_2_, NT, and NTM_2_ nanocomposites. Figure 1a shows the SEM image of pure TiO_2_ with interconnected spherical particles and a sponge-like structure, Also, the morphology of NT was appeared as spherical particles and exhibited the porous structures Fig. 1b. Apparently, the morphology of NTM_2_ is shown in Fig. 1c. MoS_2_ appeared as flowers shape grows uniformly on the surface of N-TiO_2_ spheres. TEM images of the pure TiO_2,_ NT, and NTM_2_ nanocomposites show a spherical shape with different grain sizes. Figure 1d shows the TEM micrograph of the TiO_2_ showing that the nominal size of the TiO_2_ nanoparticles is about 9 nm and that the nanoparticles appeared to be relatively homogeneous and uniform in despite being quite clustered with together. While the shape of nitrogen doped TiO_2_ is more angular and slightly longer than that of the undoped TiO_2_ with a grain size about 10 nm shown in Fig. 1e. In the case of NTM_2_, the MoS_2_ flakes appear stacked on their surface with titanium oxide in Fig. 1f. The selected area electron diffraction patterns (SAED) of T, NT and MTN_2_ respectiveley in Fig. 1g–i show that it represents the polycrystalline nature of the samples.

**Figure 2:**
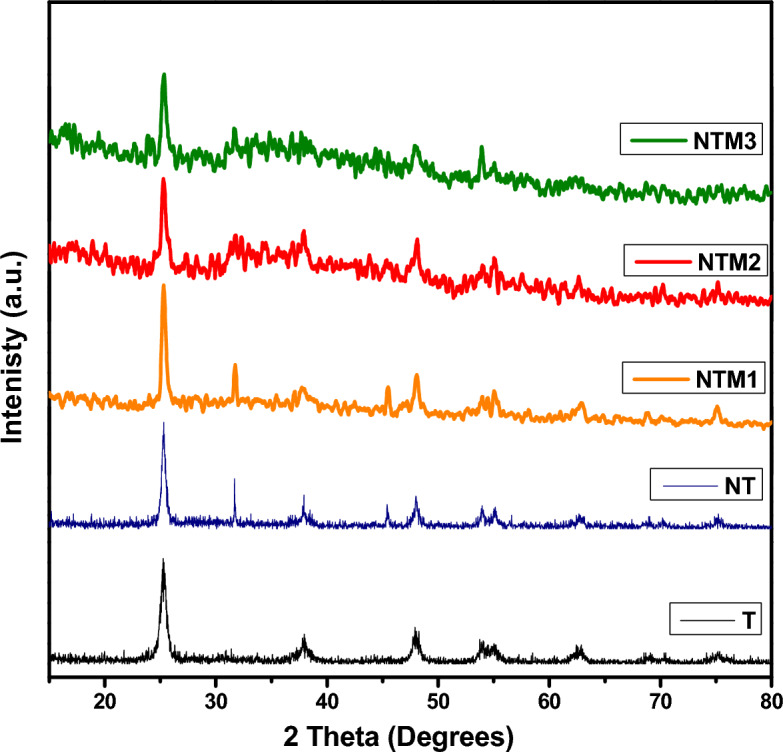
XRD pattern of T, N, NTM_1_, NTM_2_ & NTM_3_ nanocomposites.

The crystal structures of synthesized pureTiO_2_, NT, NTMs were analyzed via XRD patterns as shown in Fig. 2. Anatase (A) and rutile (R) are the two main crystalline forms of TiO_2_ commonly observed that are typically seen^36^. The XRD pattern of pure TiO_2_ shows the diffraction peaks at 2Ѳ = 20.87°, 26.65°, 36.08°,42.13°, 50.13°, and 59.96° that can be respectively matched to planes of (101), (110), (004), (111), (211), and (002), which is in good agreement to the anatase phase (JCPDS 21-1272)^37^. XRD spectrum of NT showed that nitrogen doping restricted the conversion of anatase to brookite. Also detected a peak in (1 0 1) lattice plane of anatase TiO_2_ shift to higher angles. This is attributed to the compressive stress caused by the difference in bonding properties of N and O^38^.The broad diffraction is attributed to the decrease in the grain size reduction with the destruction of crystal structure^39^. XRD spectra of the composites (NTM_1_, NTM_2_ and NTM_3_) with low and high dispersion of MoS_2_; hence no MoS_2_ diffraction peaks is seen in the spectra of NTMs composites ^38^. Moreover, compared to the composites, MoS_2_ shifts the (1 0 1) lattice plane peaks of anatase TiO_2_ to higher angles, and the peak intensity decreases with increasing MoS_2_ ratio. This is due to the large peak broadening, the peak became weaker and the affinity for amorphous structures was increased by the addition of MoS_2_^40^. This indicates that MoS_2_ is present in the NTMs composite (NTM_1_, NTM_2_ and NTM_3_).

**Figure 3:**
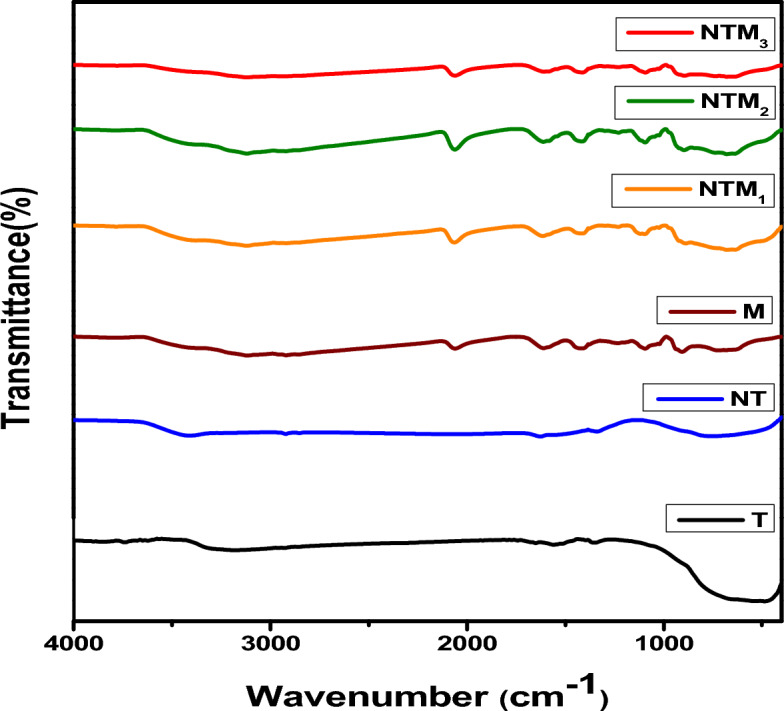
FT-IR Spectra of T, TN, M, NTM_1_, NTM_2_ and NTM_3_ nanocomposites.

Figure 3 displays the FT-IR spectra of all the samples investigated, showing strong absorption bands in the range 400–700 cm^−1^^41^. This band is assigned to the stretching vibration of the Ti–O–Ti bond. This is related to the formation of TiO_2_ and the observed shift in the composite spectra, indicating that the dopant is incorporated into the TiO_2_ lattice. The peak near 1600 cm^−1^ is attributed to the aromatics.

The C–C bond and the peak around 3400 cm^−1^ correspond to stretching vibration of OH bond. Another vibration band ranging from 1622 to 1796 cm^−1^ corresponding to the O–H bending mode was also observed. This may be attributed to the presence of H_2_O molecules adsorbed on TiO_2_^42^. The FT-IR spectrum of NT shows several absorbance peaks compared to pure TiO_2_^43^. For the MoS_2_ spectrum, the bands at 608 cm^−1^ and 1058 cm^−1^ represent the characteristic peaks of MoS_2_. These characteristic peaks of MoS_2_ are blue shifted in for NTM NCs. Therefore, the FT-IR results indicate the successful synthesis of TiO_2_, MoS_2_, and NTMs NCs ^44^.

**Figure 4:**
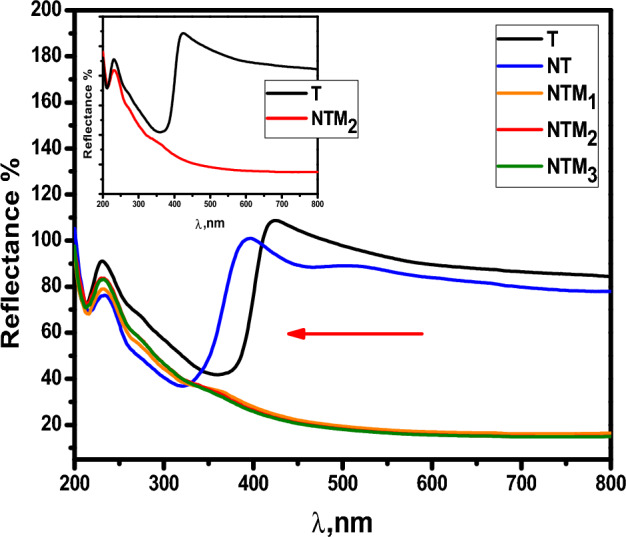
UV–Vis diffuse reflectance spectra of T, NT, NTM_1_, NTM_2_ and NTM_3_ nanocomposites;the inset shows the pure T and the optimum composite NTM_2_.

There are several mechanisms that control photocatalytic activity: electron/hole pair generation, light absorption, charge/carrier transfer, and carrier utilization. Optimization of photocatalytic activity depends on the efficiency of the products and the transfer of the e^−^/h^+^ pairs, which depends on the energy band gap (Eg) of the photocatalyst. The energy band gap value (Eg) of the samples were determined using the following Eqs.^45,46^:

1 $$\alpha hv = A\left( {hv - Eg} \right)^{n/2}$$

where α is the absorption coefficient, $v$ is the frequency of light and n is the constant of proportionality. The n value is determined by the transition of the semiconductor, i.e., the direct transition as in the prepared nanocomposite (n = 1). The diffuse reflection spectra (DRS) of T, NT, M and NTMs naocomposite were examined in the range of 200–800 nm as shown in Fig. 4. The pureTiO_2_ NP has higher absorption band edge at around 422 nm compared with the NT displayed relatively steep in absorption band edge approximately 391 nm. In the presence of MoS_2_, the band gap of the MTN_1_, MTN_2_, and MTN_3_ nanocomposites shifted toward the blue shift absorption edge around at 365 nm compared to pure TiO_2_. The addition of MoS_2_ to the TiO_2_ crystal lattice significantly increased the amount of visible light that was absorbed as a result of the effect of quantum confinement in MoS_2_ with small band gap energy (1.23 eV) which corresponds to the long wavelength absorption edge (λ = 1040 nm)^47^. As a result, the absorption edge of the composites reached to a visible region. Table 1 shows the values of the optical band gaps of the samples have been estimated from the plots of reflection percentage versus energy (hʋ), there are two band gaps for all samples. The band gap (Eg) of the composites were between 1.5 and 2 eV and approaches to 3.2 eV for the T sample. It was shown that decreasing the bandgap energy of the composite enhances the photocatalytic process by absorbing more photons and increasing the photosensitivity of the prepared photocatalyst to visible light. The stability between the e−/h+ pairs improved as a result of the conjugation of two bands gap ^2,40^.

**Table 1:** The band gap and kinetic parameters for photocatalytic activities of MB, T, M, NT, NTM_1_, NTM_2_ and NTM_3_ nanocomposites.

Sample name	E_1_ (eV)	E_2_(eV)	K_a_ (min^−1^)
MB	–	–	0.0001
T	3.4	–	0.0009
M	1.78	–	–
NT	2.89	–	0.01058
NTM_1_	1.83	3.37	0.00993
NTM_2_	1.806	3.37	0.02178
NTM_3_	1.806	3.38	0.01068

**Figure 5:**
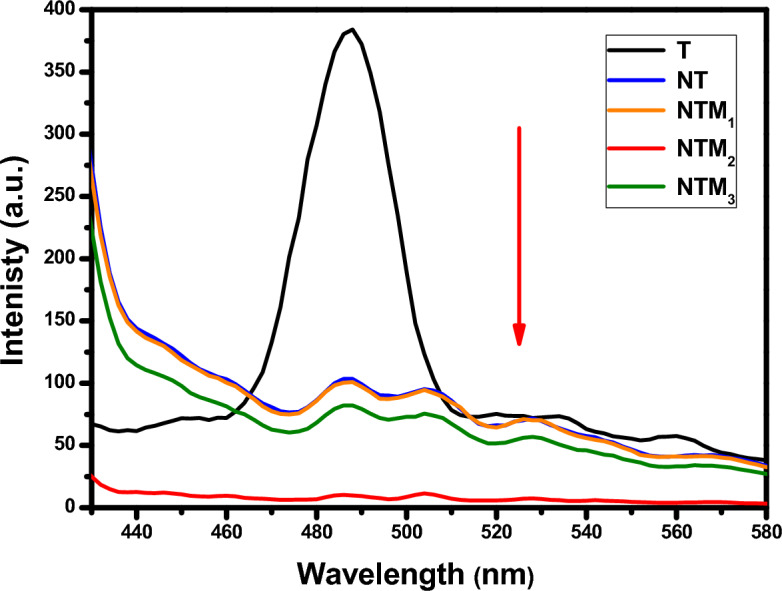
Photoluminescence spectra of T, NT, NTM_1_, NTM_2_ and NTM_3_ nanocomposites.

The room temperature photoluminescence (PL) spectra of all prepared samples are shown in Fig. 5. The PL intensity in the fluorescence emission spectra of a semiconductor photocatalyst can be used to characterize the recombination of photogenerated electrons and photogenerated holes. The lower the PL intensity of photogenerated electrons, the more effective the separation of the photogenerated cavitation. The PL spectra of T and their nanocomposites, which were found similar. The peak at ~ 390 nm is consistent with the emission of anatase TiO_2_, and peaks around at 406, 420, 445 and 480 nm for NT, NTM_1_, NTM_2_ and NTM_3_ respectively. Among all the NTM catalysts, they exhibited lowest PL intensity, and obvious fluorescence quenching, indicating that the recombination of photogenerated electrons (e−) and holes (h+) is effectively suppressed. The PL results indicate that the two-dimensional MoS_2_ layer with π-conjugated structure is an effective electron acceptor, and the separation of e−/h+ pairs under visible light irradiation is enhanced by formation of strong interactions between NT and MoS_2_ have been shown ^48,49^. A strong quenching of PL intensity in the NTM_2_ nanocomposite, indicating the recombination between the generated photoelectrons and holesis reduced.

**Figure 6:**
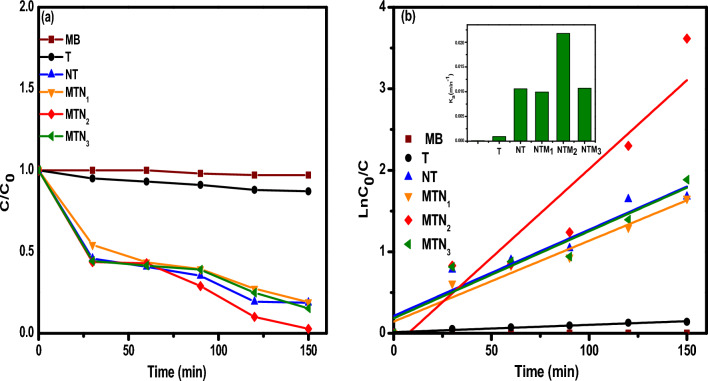
(**a**) Photodegradation and (**b**) kinetic and inset apparent rate constants for the photodegradation of MB by T, NT, NTM_1_, NTM_2_ and NTM_3_ nanocomposites.

The photocatalytic performance determined by the degradation of MB dyes under visible light induced by the pure TiO_2_, NT and their composites (MTN_1_, MTN_2_, and MTN_3_ NCs) is shown in Fig. 6a. It can be clearly seen that the NTM composite materials has a higher MB photocatalytic activity degradation of than that of TiO_2_ photocatalyst. Figure 6 measures the degradation at irradiation times of of 0, 30, 60, 90, 120 and 150 min. Before photocatalytic reaction, the photocatalyst’s MB solution was kept for 30 min in the dark to reach the adsorption/desorption equilibrium. This equation gives the efficiency of MB degradation:

2 $${\text{D }}\% \, = \, \left( {{\text{C}}_{0} - {\text{C}}/{\text{C}}_{0} } \right)*100$$

where C_0_ is the initial concentration and C is the residual concentration of MB after the reaction. The efficiency of NTM_2_ showed the highest photocatalytic degradation activity for MB dye, with a value of 99%, compared to pure TiO_2_ (13%), NTM_1_ (84.8%) and NTM_3_ (80.8%). The incorporation of MoS_2_ and N into TiO_2_ lattice in appropriate amount led to a reduction in band gap energy and sufficient PL properties, and thus, the superior photocatalytic performance of the NTM_2_ sample is directly related to it. According to the L–H kinetics model, the degradation kinetics of MB by the prepared nanocatalysts was evaluated. The pseudo-first-order kinetics equation can be expressed as:

3 $$\ln \left( {{\text{C}}_{0} /{\text{C}}} \right)\; = \;{\text{k}}_{{\text{a}}} *{\text{t}}$$

where k_a_ is the rate constant (min^−1^), C_0_ is the initial concentration (mg L^−1^), and C is the reaction concentration of the MB solution when the irradiation time is zero and t min. Figure 6b shows the relationship between ln (C_0_/C) and time. The rate constants (K_a_) can be obtained from the linear relation between them are improved in the order shown in Table 1: NTM_2_ > NTM_3_ > NTM_1_ > NT > T > MB. The NTM_2_ photocatalyst has the largest rate constant (0.02178 min^−1^) compared to TiO_2_ (0.0009 min^−1^) which is consistent with the photocatalytic degradation results, showing that the catalyst has good characteristics and and good MB degradation activity under visible light. Therefore, the prepared MTN composites can act as effective photocatalysts to degrade organic compounds with good stability. In addition, as shown in Table 2, NTM_2_ had the highest photocatalytic activity under visible light comparedto the results from previous studies.

**Table 2:** Photocatalytic degradation of MB under visible light with various photocatalysts.

Photocatalyst	Weight of catalyst (g L^−1^)	Concentration of MB (ppm)	Time (min.)	Degradation (%)	Refs.
N- TiO_2_/MoS_2_	1	10	120	98.5	^40^
N-doped TiO_2_/MoS_2_	0.01	10	100	97.9	^2^
MoS_2_–TiO_2_ NCs	0.02	25	120	61	^50^
Mos_2_/Tio_2_ Mixture	0.3	40	180	85	^51^
N-TiO_2_/AC	0.05	0.8	150	65	^52^
GCN/NT NFs	0.02	10	120	98	^53^
TiO_2_@CoFe_3_O_4_	1.0	100	60	91	^14^
N-doped TiO_2_/MoS_2_ NC_s_	0.5	50	150	99	Our work

**Figure 7:**
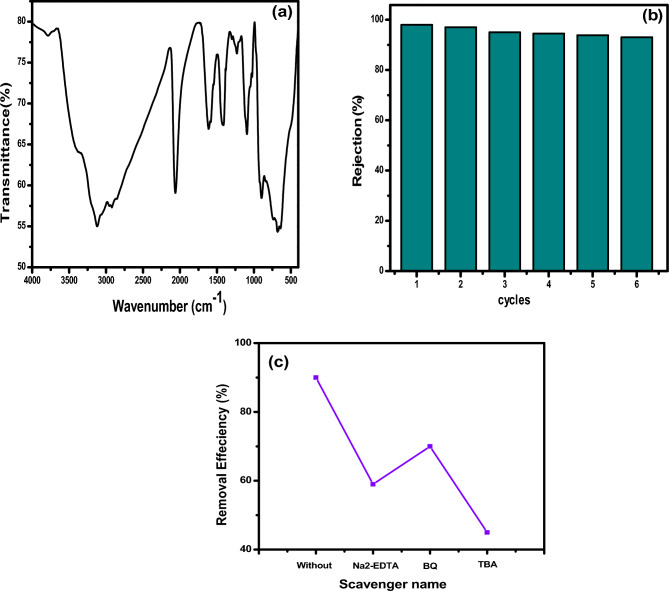
(**a**) FT-IR spectra and; (**b**) reusabilityof MTN_2_ after photocatalytic degradation for MB; (**c**) the influence of different scavengers on photodegradation efficiency of MB.

Reusability studies were examined by FT-IR after 150 min photocatalysis, as shown in Fig. 7a, the peaks perfectly correspond to the FT-IR peaks of the catalysis before the photocatalytic degradation reaction, there was no change in the peak position. These results show why, after many consecutive reuses, the prepared catalyst can maintain its catalytic efficiency as well as its stability. The degradation rate of the optimized photocatalyst NTM_2_ for MB can be recycled as shown in Fig. 7b, and its photodegradation activity was found to decrease slightly after six cycles of use, indicating that its high stability. The stability property of NTM_2_ can be attributed to the interaction between NT and MoS_2_, which can immobilize the active sites of NT nanoparticles in photocatalysis ^48^. In addition to studying the roles of free radical as shown in Fig. 7c. We applied trapping agents of free radical: Tert-butyl alcohol (TBA), p-benzoquinone (BQ) and disodium ethylene di amine tetra acetic acid (Na_2_-EDTA) to scavenge the hydroxyl radicals, superoxide radicals and holes, respectively as present in Fig. 7c. The removal efficiency of MB changed dependent on the sacrificial agents, and the removal efficiency decreased to 45% in the presence of (5 mM) TBA as hydroxyl radicals played an important role in the photodegradation of MB.

**Figure 8:**
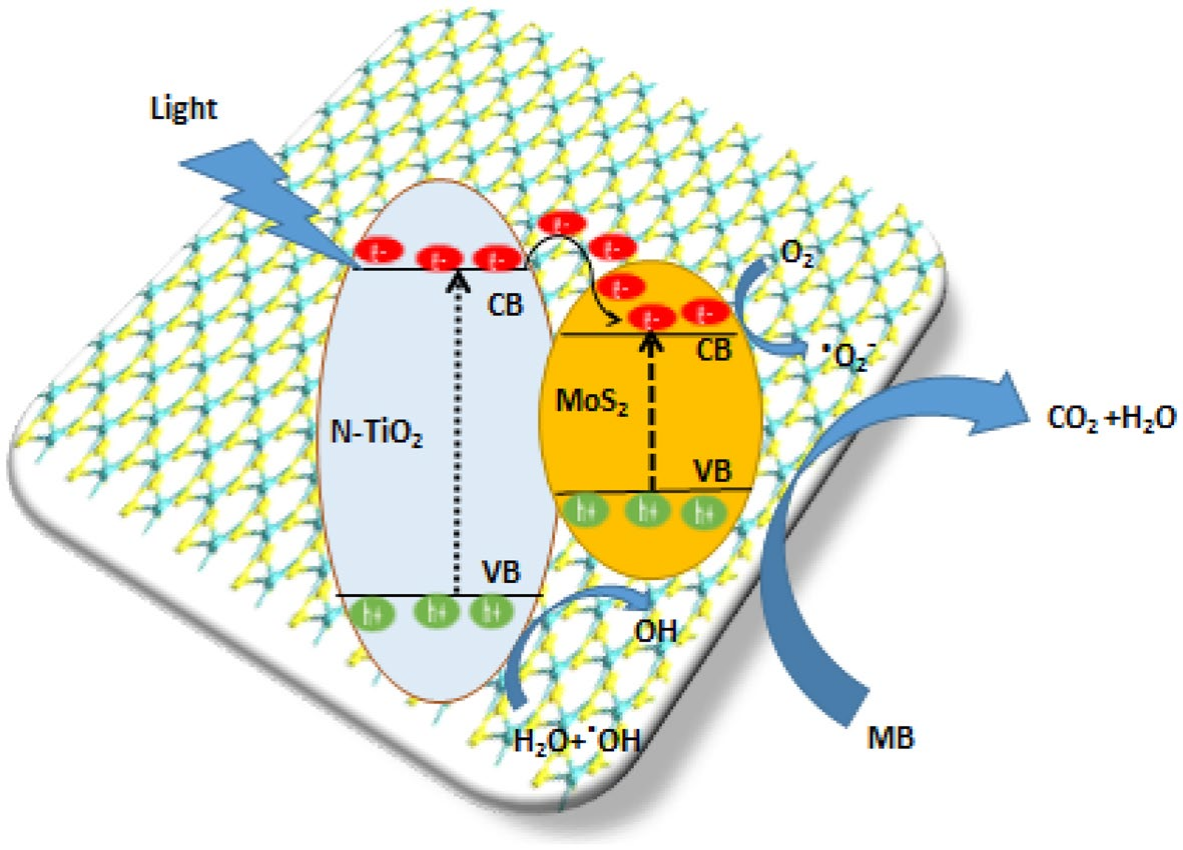
Schematic diagram of the mechanism for the photo catalytic of MB by NTM NCs under visible light irradiation.

Figure 8 illustrates the degradation mechanism based on all previous results and the energy band of hereto structure of NTMs. When N-TiO_2_ and MoS_2_ are coupled together, photons can be absorbed on the surface of the photocatalyst, resulting in the formation of electron/hole pairs. The electrons from the conduction Band (CB) of N-TiO_2_ will move to the band of MoS_2_, while holes from the valence band (VB) of N-TiO_2_ will remain there. The possibility of electron/hole recombination is reduced by this procedure. As a result, compared to NT, the NTMs composite exhibited improved photocatalytic activity. The OH^−^ in the aqueous solution is then absorbed across the hole in the valence band to give a highly reactive OH^**.**^ Radical. Finally, these active ^•^O^2^ radicals, h^+^, and ^•^OH radicals interact with MB molecules adsorbed on the surface of NTM NCs photocatalyst molecules and degrade to environmental friendly CO_2_ and H_2_O Accordingly, the increased visible-light photodegradation activity of NTMs could be due to the formation of a heterostructure between MoS_2_ and NT, which has a synergistic effect on increasing MB adsorption on the surface catalyst, improving visible light absorption improved visible light absorption, and efficient charge transport and separation. Thus, the mechanism of NTM photocatalysts after irradiated under visible light summarized by equations (Eqs. 4–10) and illustrated in schematic graph at Fig. 8 as following:

4 $${\text{NTM }} + {\text{ h}}\upsilon \; \to \; {\text{h}}^{ + } + {\text{ e}} -$$

5 $${\text{NTM}} + {\text{ O}}_{{2}} \; \to \; {\text{O}}_{{2}}^{ \cdot - }$$

6 $${\text{O}}_{{2}}^{. - } + {\text{ 2H}}_{{2}} {\text{O}}\; \to \; {\text{4OH}}^{.}$$

7 $${\text{h}}^{ + } + {\text{ H}}_{{2}} {\text{O}} \; \to \; {\text{OH}}^{.} + {\text{ H}}^{ + }$$

8 $${\text{H}}_{{2}} {\text{O }} + {\text{ H}}^{ + } + {\text{ O}}_{{2}}^{. - } \; \to \; {\text{H}}_{{2}} {\text{O}}_{{2}} + {\text{ OH}}^{.}$$

9 $${\text{H}}_{{2}} {\text{O}}_{{2}} + {\text{ e}}^{ - } \; \to \; {\text{OH}}^{ - } + {\text{ OH}}^{.}$$

10 $${\text{h}}^{ + } + {\text{ OH}}^{ - } \; \to \; {\text{OH}}^{.}$$

11 $${\text{OH}}^{.} + \;{\text{organic}}\;{\text{ pollutant }}\; \to \, \;{\text{degradation}}\;{\text{ to}}\;{\text{ CO}}_{{2}} \; + \, \;{\text{H}}_{{2}} {\text{O}}$$

## Conclusion

In summary, for more efficient MB degradation from wastewater, a nitrogen-doped titania-molybdenum sulfide (NTM) nanocomposite was used to photodegrade MB under visible light irradiation. The photocatalytic activity of NTM NCs was investigated by the degradation of methylene blue dye under visible light irradiation. It is observed that the NTM_2_ photocatalyst has a stronger visible light absorption and is seven times higher than that of pure TiO_2_. The heterostructure formation between MoS_2_ and NT, possessing the synergistic effects of enhanced MB adsorption on the catalyst surface, better visible light absorption, efficient charge transport and separation, which is highly responsible for the enhanced photodegradation activity of MB on NTMs catalyst under visible light irradiation. Reusability experiments show very high stability of NTMs. Therefore, the prepared catalyst is an excellent candidate for the efficient photocatalysis of toxic pollutants from aqueous solution.

## Data Availability

All data underlying the results are available as part of the article and no additional source date are required.
